# Supplementary material for: Dual-task costs of listening while driving in older and younger adults
Source: PLoS One. 2025 May 29;20(5):e0324657. doi: 10.1371/journal.pone.0324657 (PMC12121817; doi:10.1371/journal.pone.0324657)
Supplement: S1 Table — (DOCX) [file pone.0324657.s001.docx]

# **S1 Table**

**Total *N*’s for each demographic and assessment outcome measure.**

|  | **Older Adults** | **Younger Adults** |
| --- | --- | --- |
|  | ***N*** | ***N*** |
| **Demographics** | | |
| Age (years) | 24 | 24 |
| Education (years) | 24 | 24 |
| Driving Experience (years) | 22 | 24 |
| Self-Reported Driving Rating | 22 | 24 |
| **Hearing** | | |
| PTA better ear (dB HL) | 24 | 23 |
| CDTT SRT (dB SNR) | 24 | 24 |
| **Cognition** | | |
| MoCA | 24 | - |
| Digit Span Forward | 24 | 22 |
| Digit Span Backward | 24 | 22 |
| Digit Span Sequencing | 21 | 20 |
| Trails B-A (sec) | 22 | 24 |
| Stroop Inhibition Costs | 23 | 24 |
| UFOV Processing Speed (ms) | 22 | 23 |
| UFOV Divided Attention (ms) | 22 | 23 |
| UFOV Selective Attention (ms) | 22 | 23 |
| **Vision** | | |
| ETDRS Left Eye (logMAR) | 24 | 24 |
| ETDRS Right Eye (logMAR) | 24 | 24 |

PTA = Pure tone average, dB HL = decibel hearing loss. CDTT SRT = Canadian Digit Triplet Test Speech Reception Threshold, dB SNR = decibel signal-to-noise-ratio. MoCA = Montreal Cognitive Assessment. ETDRS = Early Treatment Diabetic Retinopathy Study. UFOV = Useful Field of View.
