# Supplementary material for: Dual-task costs of listening while driving in older and younger adults
Source: PLoS One. 2025 May 29;20(5):e0324657. doi: 10.1371/journal.pone.0324657 (PMC12121817; doi:10.1371/journal.pone.0324657)
Supplement: S2 Fig — (DOCX) [file pone.0324657.s002.docx]

**S2 Figure**


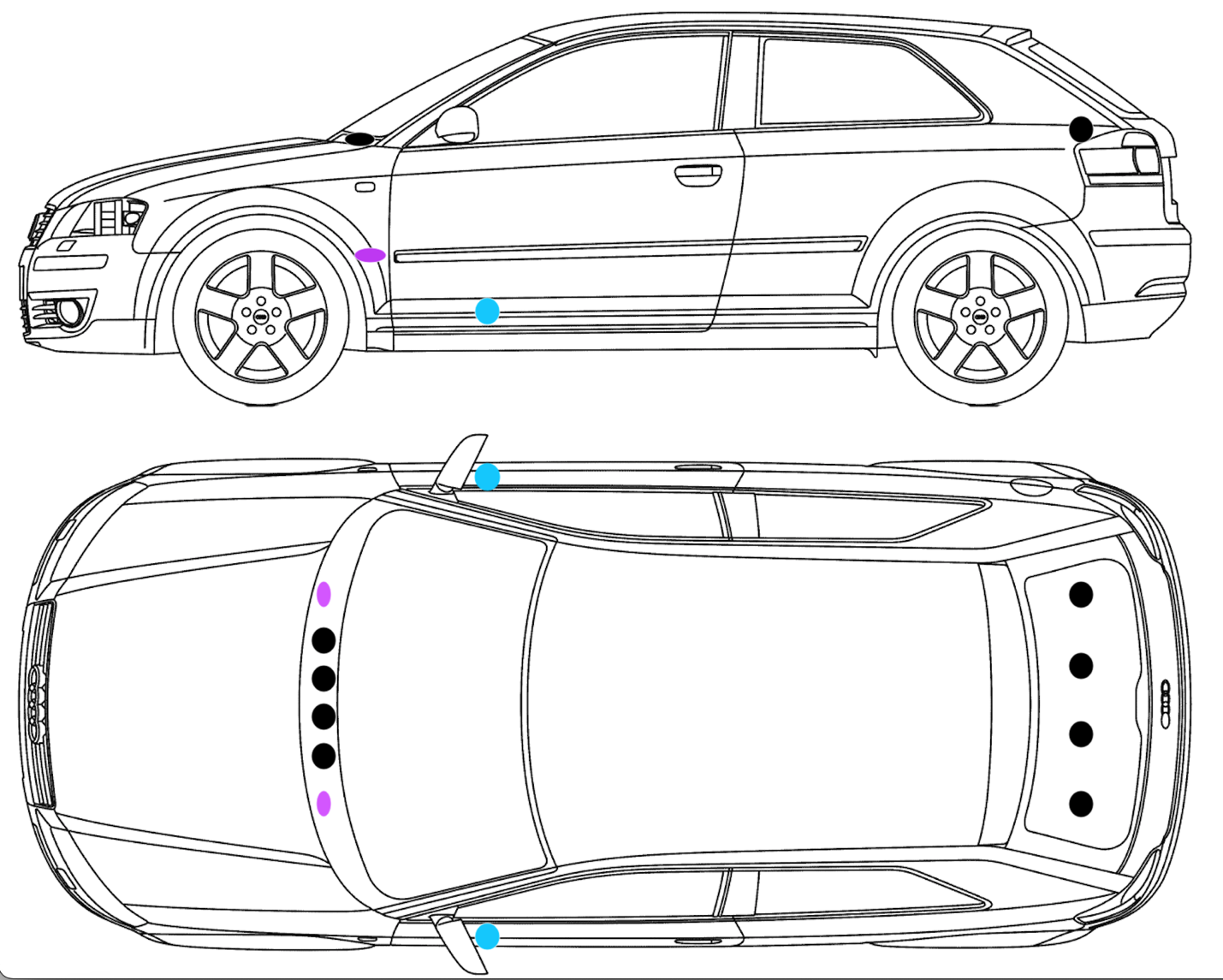


**Figure S2. Side-view and top-down view of the speaker configuration in DriverLab.** The top view shows the 6 loudspeakers located in front of the participant (4 black dots and 2 purple dots at the front of the vehicle dashboard), 2 loudspeakers on either side of the participant (2 blue dots), and 4 loudspeakers in the trunk of the vehicle (4 black dots at the back of the vehicle). The side-view shows the relative vertical position of the same colour coded speakers.
