# Supplementary material for: Dual-task costs of listening while driving in older and younger adults
Source: PLoS One. 2025 May 29;20(5):e0324657. doi: 10.1371/journal.pone.0324657 (PMC12121817; doi:10.1371/journal.pone.0324657)
Supplement: S3 Fig — (DOCX) [file pone.0324657.s003.docx]

**S3 Figure**


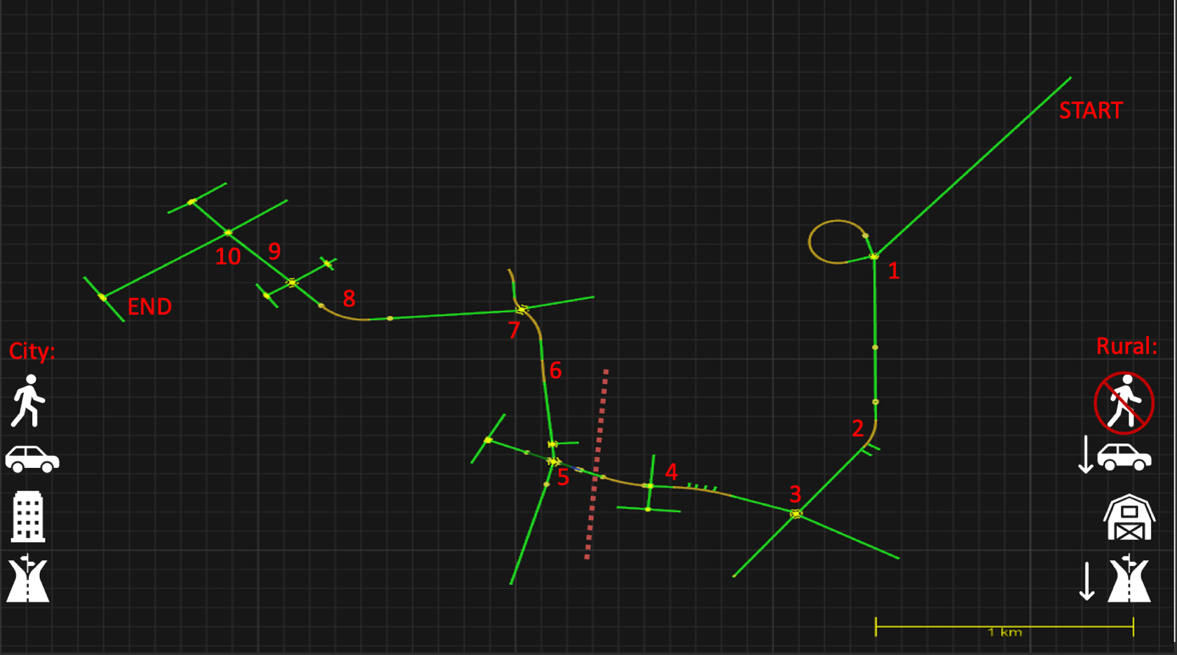


**Figure S3. Top-down view of a scenario map.** Top-down view of the +4 SNR dual-task driving scenario map. Roadway to the right of the red dotted line includes the rural section of the drive. Roadway to the left of the red dotted line includes the city section of the drive. Rural: 1) left-hand turn, 2) a gradual curve, 3) right-hand turn, and 4) a stopped car. City: 5) right-hand turn, 6) pedestrian crosswalk, 7), left-hand turn, 8) gradual curve, 9) construction zone, 10) left-hand turn.
