# Supplementary material for: Dual-task costs of listening while driving in older and younger adults
Source: PLoS One. 2025 May 29;20(5):e0324657. doi: 10.1371/journal.pone.0324657 (PMC12121817; doi:10.1371/journal.pone.0324657)

**S4 File**

**Listening performance – Proportional dual-task costs (accuracy)**

With regards to proportional dual-task costs to listening performance, there were significant main effects of Age Group (*F*(1, 46) = 4.15, *p* = 0.047, *η²* = .03), Driving Difficulty (*F*(1, 46) = 4.92, *p* = 0.032, *η²* = .02), and Listening Difficulty (*F*(1, 46) = 8.99, *p* = 0.004, *η²* = .05) but no significant two-way interaction effects (*p* > 0.05). There was a three-way Age Group x Driving Difficulty x Listening Difficulty interaction effect that approached significance and thus, should be interpreted with caution (*F*(1, 46) = 3.79, *p* = 0.058, *η²* = .02). Post-hoc analyses for this three-way interaction can be found in the main document (see Fig S4 below).

## **Determining whether proportional dual-task costs differ from zero (listening performance accuracy)**

One sample t-tests were conducted to examine whether proportional dual-task effects significantly differed from zero. Since these t-tests are independent from each other, no Bonferroni correction was used. One sample t-tests to examine significant dual-task costs/benefits revealed that for the older adult group, proportional dual-task costs were observed in the City sections under both the 0 dB SNR (*t*(23) = 5.83, *p* < 0.0001) and the +4 dB SNR Listening Condition, (*t*(23) = 2.10, *p* < 0.05), as well as in the Rural sections under the +4 dB SNR Listening Condition, (*t*(23) = 2.27, *p* < 0.05), but not the 0 dB SNR Listening Condition, (*p* > 0.05). For younger adults, proportional dual-task benefits were observed in the Rural section under the +4 dB SNR Listening Condition, (*t*(23) = -2.55, *p* < 0.05); however, no proportional dual-task costs were observed in any other conditions (*p* > 0.05) (see Fig S4 below).

**Fig S4. Proportional dual-task costs (DTC) to listening performance accuracy scores, for each Age Group, Listening Difficulty, and Driving Difficulty condition.** Positive values indicate a dual-task cost (poorer performance in Dual compared to Single Task). Each violin plot represents the frequency of the data at each point on the y-axis. The center dot represents the mean. Error bars represent ±1 SE. Large red asterisks represent significant differences relative to zero, with their position above or below the plot indicating whether they represent dual-task costs or benefit. Small black asterisks represent *p*-values: * = *p* < 0.05.


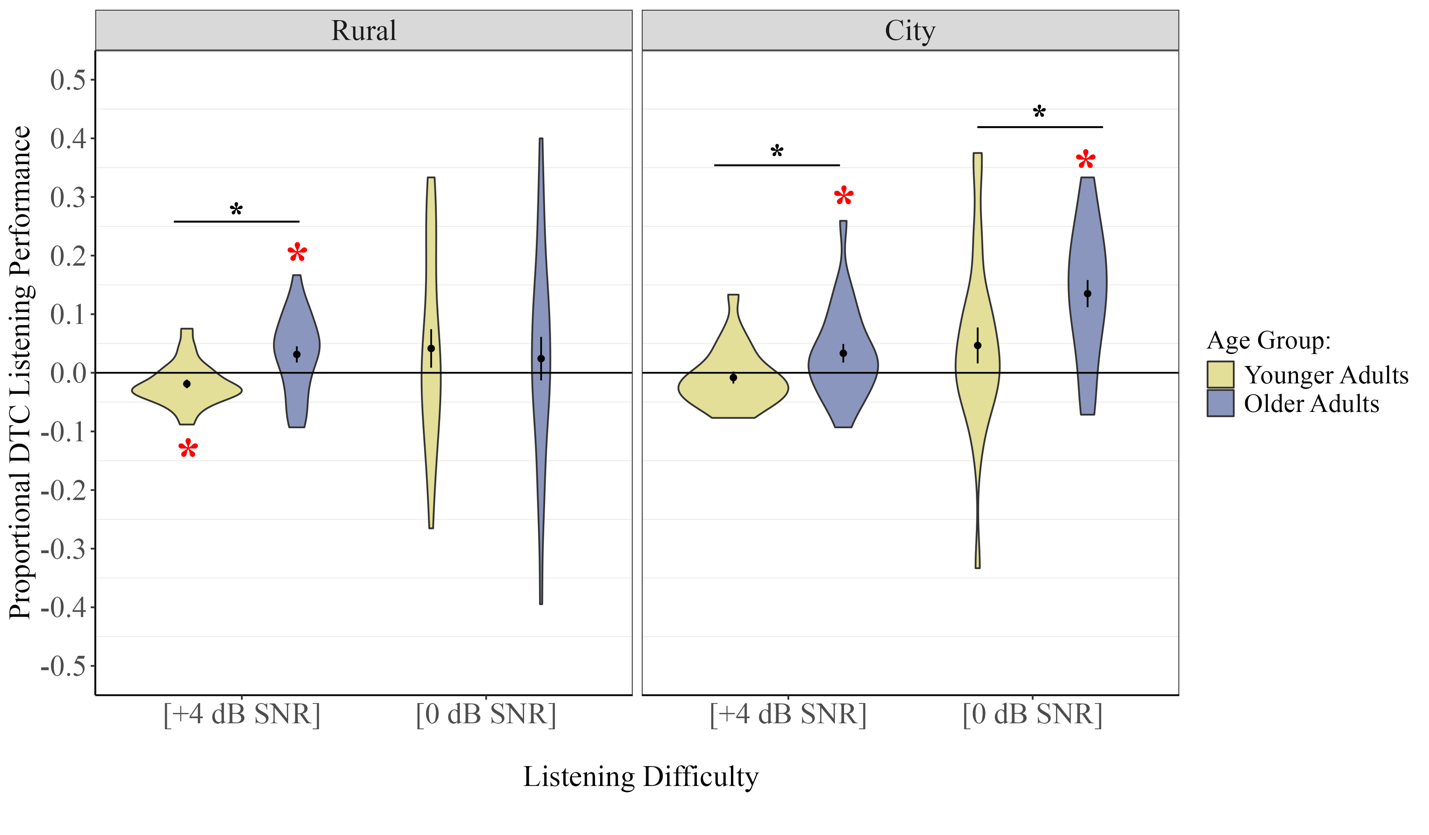

Supplement: S4 File — (DOCX) [file pone.0324657.s004.docx]
