# Supplementary material for: Dual-task costs of listening while driving in older and younger adults
Source: PLoS One. 2025 May 29;20(5):e0324657. doi: 10.1371/journal.pone.0324657 (PMC12121817; doi:10.1371/journal.pone.0324657)
Supplement: S5 File — (DOCX) [file pone.0324657.s005.docx]

**S5 File**

**Driving performance – Proportional dual-task costs (standard deviation of lane position)**

For proportional dual-task costs to driving performance, significant main effects of Driving Difficulty (*F*(1, 46) = 39.49, *p* < 0.001, *η²* = .20) and Listening Difficulty (*F*(1, 46) = 9.10, *p* = 0.004, *η²* = .03) were observed. Significant two-way interaction effects of Age Group x Driving Difficulty and Listening Difficulty x Driving Difficulty were observed (*p* < 0.05), which were further qualified by a significant three-way Age Group x Driving Difficulty x Listening Difficulty interaction effect, *F*(1, 46) = 7.47, *p* = 0.009, *η²* = .01. Post-hoc comparisons revealed that, in the City section, older adults had significantly higher proportional dual-task costs to SDLP in the 0 dB SNR Listening Difficulty compared to the +4 SNR Listening Difficulty condition, *t*(46) = -4.55, *p* < 0.001; however, proportional dual-task costs to SDLP were not significantly different between Listening Difficulty conditions for younger adults in the City section, nor were there any significant differences between proportional dual-task costs to SDLP in the Rural section for either Age Group (all comparisons *p* > 0.05). Additional post-hoc comparisons were conducted and can be found in the main document (see Fig S5 below).

## **Determining whether proportional dual-task costs differ from zero (driving performance standard deviation of lane position [SDLP])**

One sample t-tests were conducted to examine whether proportional dual-task effects significantly differed from zero. Since these t-tests are independent from each other, no Bonferroni correction was used. One sample t-tests to examine significant dual-task costs/benefits revealed that for older adults, significant proportional dual-task costs occurred only in the City section under both the 0 dB SNR (*t*(23) = 8.88, *p* < 0.0001) and the +4 dB SNR Listening Conditions (*t*(23) = 3.55, *p* < 0.01) but not in the Rural sections (*p* > 0.05). For younger adults, significant proportional dual-task costs only occurred in the City section under both the 0 dB SNR (*t*(23) = 4.36, *p* < .001) and +4 dB SNR Listening Conditions, (*t*(23) = 3.77, *p* < 0.05), but not the Rural sections (*p* > 0.05) (see Fig S5 below).


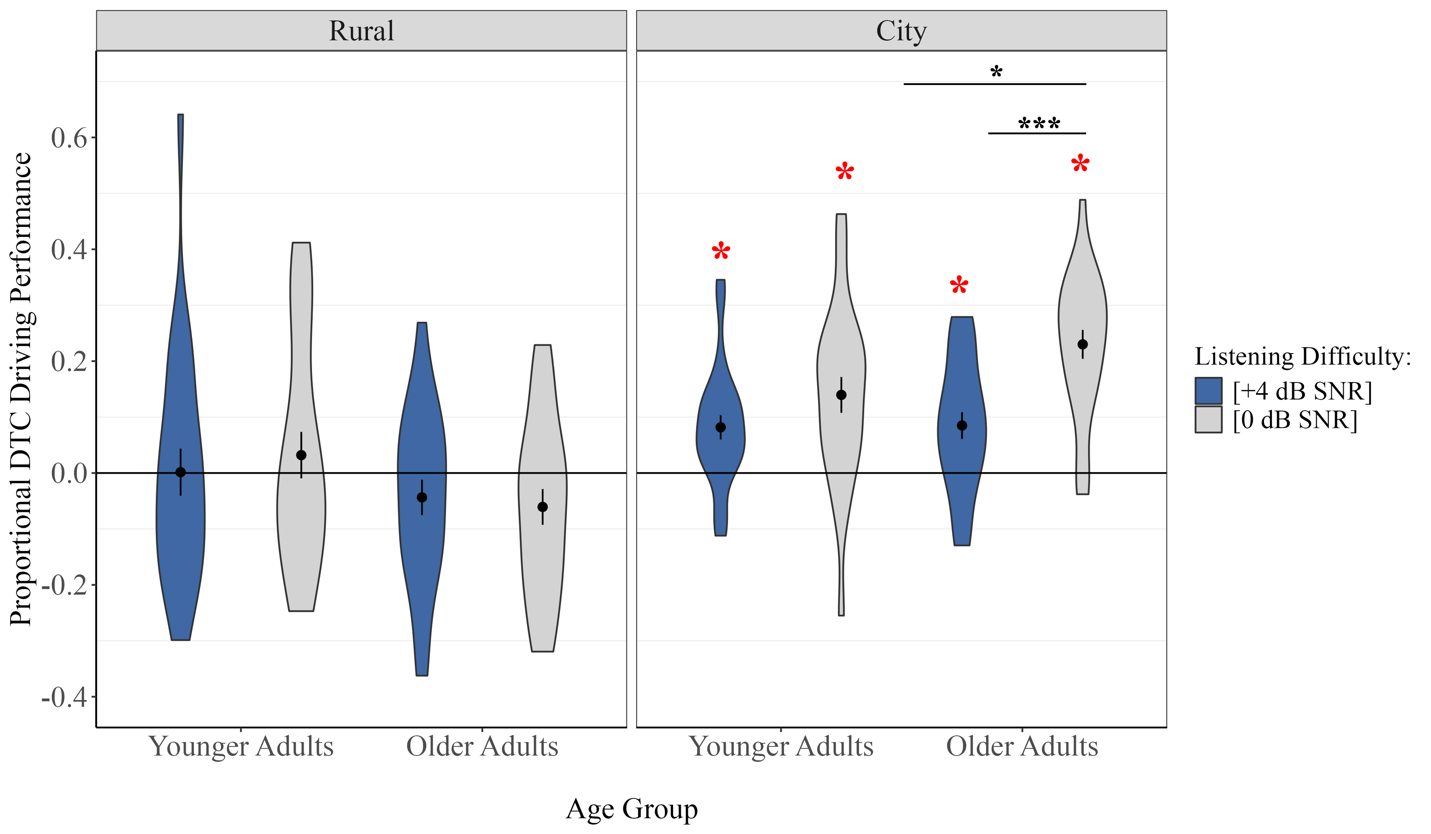


**Fig S5. Proportional dual-task costs (DTCs) to SDLP, for each Age Group, Listening Difficulty, and Driving Difficulty condition.** Each violin plot represents the frequency of the data at each point on the y-axis. The center dot represents the mean. Error bars represent ±1 SE. Positive values indicate a dual-task cost (poorer performance in Dual-Task compared to Single-Task performance). Large red asterisks represent significant differences relative to zero, with their position above or below the plot indicating whether they represent dual-task costs or benefit. Small black asterisks represent *p*-values: * = *p* < 0.05, *** = *p* < 0.001.
