# Supplementary material for: Dual-task costs of listening while driving in older and younger adults
Source: PLoS One. 2025 May 29;20(5):e0324657. doi: 10.1371/journal.pone.0324657 (PMC12121817; doi:10.1371/journal.pone.0324657)
Supplement: S6 File — (DOCX) [file pone.0324657.s006.docx]

**S6 File**

**Associations among sensory and cognitive baseline measures**

Pure tone average (PTA) in the better ear and speech reception thresholds measured using the Canadian Digit Triplets Test (CDTT SRT) was positively correlated, demonstrating that better hearing was associated with better speech-in-noise thresholds. Early Treatment Diabetic Retinopathy Study (ETDRS) was positively correlated with the Useful Field of View (UFOV) Divided and Selective Attention, but not UFOV Processing Speed, suggesting that better visual acuity is associated with better visual divided and selective attention.

PTA and CDTT SRT were not correlated with standard cognitive measures of working memory (Digit Span Total), inhibition (Stroop Inhibition Costs), and task switching (Trail Making). However, PTA and CDTT SRT were positively correlated with almost all UFOV measures, with the strongest correlation being with UFOV Selective Attention and PTA. UFOV, particularly the Selective Attention subtest, has been associated with driving performance in past literature. Similarly, the cognitive measure of task switching (Trail Making) was positively correlated with UFOV Divided Attention, suggesting that better task switching ability was
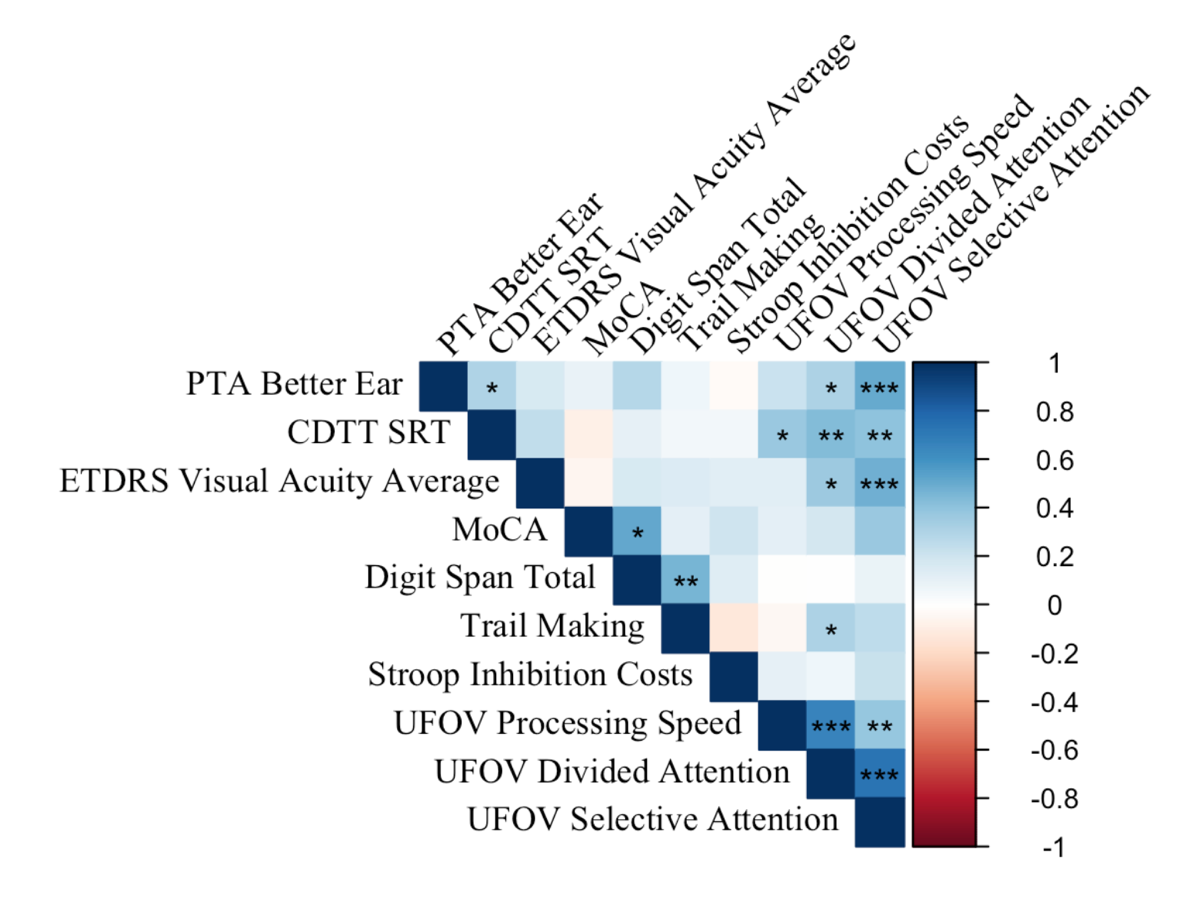
associated with better visual divided attention.

**Figure S6. Correlations between baseline measures.** To ensure consistency, PTA in the better ear, Trail Making scores, UFOV Processing Speed, Divided Attention, and Selective Attention sub-tests scores were reverse coded so that higher values indicated better performance across all baseline measures. All CDTT SRT values are absolute values. A positive r value indicates a positive relationship (blue) and a negative r value indicates a negative relationship (red). * = *p* < 0.05, ** = *p* < 0.01, *** = *p* < 0.001.
