# Supplementary material for: Dual-task costs of listening while driving in older and younger adults
Source: PLoS One. 2025 May 29;20(5):e0324657. doi: 10.1371/journal.pone.0324657 (PMC12121817; doi:10.1371/journal.pone.0324657)
Supplement: S7 File — (DOCX) [file pone.0324657.s007.docx]

**S7 File**

**Relative task costs between listening and driving performance in the rural sections**

A series of paired samples t-tests were conducted to compare proportional dual-task costs to listening performance to proportional dual-task costs to driving performance separately for each age group in the rural section. Since these t-tests are independent from each other, no Bonferroni correction was used. For older adults, results were approaching significance for proportional dual-task costs to standard deviation of lane position (SDLP) compared to proportional dual-task costs to listening accuracy in the Rural 0 dB SNR Listening Condition, *t*(23) = 1.99, *p* = 0.058, and in the Rural +4 dB SNR Listening Condition, *t*(23) = -2.00, *p* = 0.057. These findings should be interpreted with caution. However, for younger adults, results were not significantly different between proportional dual-task costs to SDLP and listening accuracy in the Rural section for either Listening Condition (*p* > 0.05).
