# Supplementary material for: Dual-task costs of listening while driving in older and younger adults
Source: PLoS One. 2025 May 29;20(5):e0324657. doi: 10.1371/journal.pone.0324657 (PMC12121817; doi:10.1371/journal.pone.0324657)
Supplement: Attachment — Submitted filename: Response to Reviewers.docx [file pone.0324657.s009.docx]

We would like to sincerely thank the editor and reviewers for taking the time to provide such thoughtful comments towards improving our manuscript. This document lists all comments specific to each reviewer, interleaved with the authors’ responses and, when applicable, references to the changes made to the manuscript. We are confident that these changes have improved the manuscript and we hope that you will again consider it for publication in PLOS ONE.

**1. Responses to Journal Requirements:**

*1.1. Please ensure that your manuscript meets PLOS ONE's style requirements, including those for file naming. The PLOS ONE style templates can be found at*

[*https://journals.plos.org/plosone/s/file?id=wjVg/PLOSOne_formatting_sample_main_body.pdf*](https://journals.plos.org/plosone/s/file?id=wjVg/PLOSOne_formatting_sample_main_body.pdf) *and*

[*https://journals.plos.org/plosone/s/file?id=ba62/PLOSOne_formatting_sample_title_authors_affiliations.pdf*](https://journals.plos.org/plosone/s/file?id=ba62/PLOSOne_formatting_sample_title_authors_affiliations.pdf)

**Response:** Thank you for the prompt. We have double checked that the manuscript complies with PLOS ONE’s style requirements.

*1.2. We note that you have indicated that there are restrictions to data sharing for this study. PLOS only allows data to be available upon request if there are legal or ethical restrictions on sharing data publicly. For more information on unacceptable data access restrictions, please see* [*http://journals.plos.org/plosone/s/data-availability#loc-unacceptable-data-access-restrictions*](http://journals.plos.org/plosone/s/data-availability#loc-unacceptable-data-access-restrictions)*. If there are ethical or legal restrictions on sharing a de-identified data set, please explain them in detail (e.g., data contain potentially identifying or sensitive patient information, data are owned by a third-party organization, etc.) and who has imposed them (e.g., a Research Ethics Committee or Institutional Review Board, etc.). Please also provide contact information for a data access committee, ethics committee, or other institutional body to which data requests may be sent.*

**Response:** Thank you for the additional information regarding data sharing restrictions. We have obtained a formal letter from our Research Ethics Board which explains in detail the ethical restrictions on sharing a de-identified dataset. Please see the document titled “UHN REB Letter on Sharing De-Identified Data” in the PLOS ONE portal. For your reference, the body of the letter states, “I am writing in my capacity as the Board Lead for the Research Ethics Board (REB) at the University Health Network and REB coordinator of the REB file regarding the manuscript titled “Dual-task costs of listening while driving in older and younger adults,” authored by Katherine Bak, Kristen Arnold, Lena Darakijan, M. Kathleen Pichora-Fuller, Frank A. Russo, and Jennifer L. Campos. The protocol and consent form for this study, as approved by our REB, specifically restrict the sharing of de-identified data publicly. This restriction is based on the language included in the consent process, which explicitly states that data collected from participants will only be accessible to and shared within the study team. Consequently, sharing the de-identified dataset with external entities, such as other researchers or organizations, would constitute a breach of the conditions under which participant consent was obtained. The only exception to this is if sharing of this data is required by law. Even if sharing were possible, the data that UHN REB would allow would be extremely limited due to the nature of the information collected for the study. Such REB restrictions would be in place to minimize any potential risks of re-identification or misinterpretation outside of its intended context. UHN REB recognizes that such limitations would inherently impact the reproducibility of any analyses derived from the dataset. These restrictions were imposed by The University Health Network's Research Ethics Board, an independent body responsible for ensuring compliance with ethical standards and participant protections. Any requests for further information or clarifications about the study's data-sharing policies may be directed to our REB office.”

**Contact Information for the REB:**

Research Ethics Board
University Health Network
700 University Ave. 4^th^ floor
Toronto, ON, M5G 1Z5
Email: [reb@uhn.ca](mailto:reb@uhn.ca)
Phone: 416-581-7849 (main line)

Thank you for your understanding of the ethical considerations that underpin our decision-making in this matter. If you require further clarification, please do not hesitate to contact me or the REB directly.

Sincerely,

*Lorraine Pirrie*

*1.3. Please review your reference list to ensure that it is complete and correct. If you have cited papers that have been retracted, please include the rationale for doing so in the manuscript text, or remove these references and replace them with relevant current references. Any changes to the reference list should be mentioned in the rebuttal letter that accompanies your revised manuscript. If you need to cite a retracted article, indicate the article’s retracted status in the References list and also include a citation and full reference for the retraction notice.*

**Response:** Thank you for the prompt. The reference list has been reviewed and is complete and correct. URLs were added where relevant.

Note that we removed the Suen et al (2021) reference for asymmetrical hearing loss criteria, as the Editor kindly pointed out that the main purpose of this paper is to report on the prevalence of asymmetrical hearing loss for different criteria. As such, we have added the correct reference to the reference list (American Academy of Otorhinolaryngology- Head and Neck Surgery [AAO-HNS]. Position Statement: Red Flags-Warning of Ear Disease, 2021).

We also added the Rothman 1990 reference to support the rationale for not correcting for multiple comparisons in our correlations plots (“Rothman K. J. (1990). No adjustments are needed for multiple comparisons. *Epidemiology (Cambridge, Mass.)*, *1*(1), 43–46.”).

**2. Responses to Editor Comments:**

*2.1. Did any of the participants were multilingual? If yes, do the authors think that it could*

**Response:** Thank you for this insightful comment. We did not specifically ask for multilingualism; however, we did ask for participants’ primary language of choice. All participants who responded to this question stated that English was their primary language of choice, except for two participants who stated Persian (1 younger adult) and Farsi (1 older adult) as their primary language of choice. We have added these additional details to the manuscript (Page 5 lines 156-158) which now reads *“All participants provided written informed consent, spoke fluent English, learned English by the age of, 5 and* *reported English as their primary language of choice (with the exception of one younger adult and one older adult participant)…”* Please also see section 3, responses to Reviewer #1, Comment 3.11.

*2.2. Lines 164-169: Did the authors screen hearing or did obtain hearing thresholds? Hearing screening is conducted usually at one intensity level (usually 25 dB HL).*

**Response:** We did not do a hearing screening at one intensity level. Instead, we screened hearing based on audiometric pure-tone air-conduction thresholds obtained using ShoeBox^TM^ (see the detailed procedures on the pure-tone audiometry on Page 6 lines 171-174). We have updated the manuscript to further clarify what kind of hearing test was completed. Page 6 lines 171-174 now reads “*A screening hearing test was administered in-person using Shoebox^TM^ (Version 5.6.7); participants’ pure-tone audiometric air-conduction thresholds (dB HL) were measured in each ear across five frequencies of 500 Hz, 1000 Hz, 2000 Hz, 3000 Hz, and 4000 Hz to determine eligibility*.” We describe this as a screening because we obtained pure-tone thresholds for the purposes of our research, but we did not conduct full audiometry for diagnostic purposes (e.g., testing did not include bone-condition or masked thresholds).

*2.3. Why was hearing not tested at 6 and 8 kHz? Hearing loss at these frequencies is very common in older adults. This could affect speech recognition in difficult listening conditions.*

**Response:** Thank you for this comment. Our ShoeBox^TM^ hearing test did measure pure-tone audiometric thresholds (dB HL) in each ear across the frequencies of 6000 Hz and 8000 Hz, in addition to the 500 Hz, 1000 Hz, 2000 Hz, 3000 Hz, and 4000 Hz thresholds used for eligibility criteria. We used the frequencies of 500 Hz, 1000 Hz, 2000 Hz, 3000 Hz, and 4000 Hz as these frequencies are used to classify individuals into normal hearing and hearing loss categories, as per the World Health Organization (WHO) criteria. We have added a plot of hearing thresholds across the frequencies of 500 Hz to 8000 Hz for each group. Please also see our response to comment 2.4 below.

*2.4. I would plot hearing thresholds for each test frequency for both groups.*

**Response:** Thank you for the helpful suggestion to include a plot of hearing thresholds for each test frequency for both groups. We have added an additional figure of hearing thresholds at each frequency (500 Hz – 8000 Hz) for both groups, which will be inserted on Page 7 of the Methods section, at the end of the Hearing subsection (please also see below for new figure).

**Fig 1*.* Hearing thresholds at each frequency (500 Hz – 8000 Hz) and ear for both younger and older adult groups.** The circle and triangle symbols represent the means. Error bars represent ±SE.

*2.5. Lines 169-171: 15-dB interaural difference was recommended by the American Academy of Otorhinolaryngology- Head and Neck Surgery (*[*https://www.entnet.org/resource/position-statement-red-flags-warning-of-ear-disease/*](https://www.entnet.org/resource/position-statement-red-flags-warning-of-ear-disease/)*). Suen et al (2021) reported the prevalence of asymmetrical hearing loss for different criteria (10-, 15- and 20-dB HL).*

**Response:** Thank you for pointing out the correct citation. We have updated the reference to include the American Academy of Otolaryngology-Head and Neck Surgery (AAO-HNS) online statement which defines asymmetrical hearing loss using the criteria outlined in the manuscript (Page 6, line 178).

*2.6. Line 171: PTA means pure tone average or pure tone thresholds.*

**Response:** Thank you for pointing out that we did not clearly state what PTA stands for. We have added “pure-tone threshold average” before the first mention of PTA in the manuscript (Page 6, line 178).

*2.7. Lines 172-175: The authors should provide more details about the digit triplet test. How was it administered? Was this test online?*

**Response:** Thank you for pointing out that we did not clearly state what the digit triplet test is measuring and whether it was tested in-person or online. We have updated the manuscript to state that the CDTT was conducted in-person and that it is a speech-in-noise test that measures speech reception thresholds in noise (Page 6, line 181-182). We also note that the digit triplet test was administered as per the procedures outlined in reference 33.

*2.8. There are no sufficient details about the calibration. How did the authors measure the sound levels in the DriverLab environment? How far were the speakers from the listener?*

**Response:** The REED R8050 Sound Level Meter (SLM) was used to measure the baseline noise level (when the car was turned on and parked) and the average background noise level (including the CST babble and DriverLab environmental and vehicle noises (e.g., engine noises, tires rumbling on the pavement, A/C fans)) at head level when seated (Page 10 line 270-271, and Page 11 lines 305 – 307). The SLM was first calibrated using the 1⁄2” pre-polarised microphone (4230 pistonphone calibrator) to produce a 1000-Hz pure tone at 94 dB SPL in free field while sitting in DriverLab. These details regarding calibration have been added to the manuscript on Pages 10 lines 271- 272 and Page 11 line 288 - 289. Please also see response to comment 2.9 below.

The distance of the loudspeakers and the speaker configuration has also been updated in the manuscript on Page 10, lines 262 to 270. Additionally, a diagram of the speaker locations has been included in the supplementary materials (please also see below for new figure). In total there were twelve loudspeakers. Specifically, six loudspeakers are located in front of the participant (~24 inches in front of the driver’s seat), four in the middle of the dashboard, one below the dashboard above the driver’s feet, and one below the dashboard above the passenger’s feet (~39 inches to the right of the driver’s seat). Additionally, one loudspeaker is in the driver’s side door (~12 inches in front of the driver’s seat) and one loudspeaker in the front passenger side door (~12 inches in front and 48 inches to the right of the driver’s seat). Four loudspeakers are also in the trunk of the vehicle (~68 inches behind the driver’s seat) (See S2).

**S2 Figure. Side-view and top-down view of the speaker configuration in DriverLab.**

*2.9. Lines 251-252: How was the sound level was measured? Did sound levels vary based on different driving scenarios? Depending on the scenario, should the background noise levels vary?*

**Response:** As stated in our response to comment 2.8 above, sound level measurements were obtained using the REED R8050 SLM (which was calibrated before each use) from the position of the head when seated. The average baseline noise level and background noise level were measured, and the CST signal was presented at a +4 dB SNR and a 0 dB SNR as stated in the manuscript. Each driving scenario contained the same driving elements and tasks (e.g., same curves, same number of turns at the same angle, etc.), but in a different order. As such, sound levels were on average consistent across drives. We would also like to note that the background driving environmental noise levels (e.g., variations due to acceleration, speed) may have varied across a scenario and/or between participants depending on how the vehicle was maneuvered. For example, one participant may have accelerated more quickly during a straightaway compared to another participant who may have accelerated more slowly, causing an increase in acceleration-related noise levels. However, we are confident that any potential differences in environmental noise levels across participants and scenarios were minimal as, 1) participants were required to follow a lead car which limited fluctuations in speed throughout the scenarios, and 2) participants never had to bring the car to a full stop, as yield signs were used instead of stop signs at intersections, which limited large fluctuations in acceleration throughout the scenarios. In addition to adding information about obtaining sound measurements using the REED R8050 SLM, we have also added that sound levels were on average consistent across drives (Page 11 lines 303-304).

*2.10. Line 259: Regarding the connected speech test (CST), why didn’t the author use the latest version of the CST (Saleh et al., 2020)? The older version of the CST has a higher noise floor (Saleh et al., 2020) and the accent differed. How will the authors justify the noise floor in the CST recording and its effect on the results of the current study? Saleh HK, Folkeard P, Macpherson E, Scollie S. Adaptation of the Connected Speech Test: Rerecording and Passage Equivalency. Am J Audiol. 2020 Jun 8;29(2):259-264. doi: 10.1044/2019_AJA-19-00052. Epub 2020 Mar 20. PMID: 32196353.*

**Response:** Thank you for bringing this important paper to our attention. Methodological considerations for this study were finalized in 2019, before publication of this latest version of the CST. We also conducted a follow-up study with older adults with and without age-related hearing loss, which uses the same methodology described in this manuscript. The original CST was adapted by grouping topics to ensure equivalency, addressing differences observed in individuals with hearing loss when using the original topic set. To our knowledge, the latest version of the CST has not yet been tested in those with hearing loss. Therefore, to maintain consistency across our two studies, we chose to use the original CST with topic equivalency for both individuals with normal hearing and hearing loss. Additionally, the latest CST development paper did not compare intelligibility scores between the original test materials and their new test materials. As such, it is unclear whether the differences in accent and noise floor yield significant differences in intelligibility scores.

We would also like to note that there is a noticeable southern accent for a few of the words in some topics, as you have noted. However, we feel any potential effects on intelligibility would be minimal, due to the very few instances these words come up. Furthermore, every participant heard the same sentences, so any potential effects on intelligibility would be relative across groups and conditions. Similarly, a higher noise floor would affect all sentences equally, and since all participants heard the same sentences, any potential impact of the higher noise floor would remain consistent across groups and conditions. We would finally like to note that the DriverLab environment differs from the highly controlled settings typically used in other studies employing the CST. Unlike the quiet and highly controlled sound booths commonly used, DriverLab is a reverberant, free field setting with background noises such as engine sounds and fans. As such, potential effects of the noise floor may be less apparent in the current study compared to the typical highly controlled studies.

*2.11. Provide information about the scoring for the CST.*

**Response:** We did not go into detail about the scoring of the CST in the methods section until the data analyses section of the manuscript, which we can appreciate may have caused some confusion. Therefore, in the revised version, upon first mention of the CST in the Method section, we have added in brackets “*see Data Analyses section below for information about scoring of the CST”* (Page 11 lines 302 - 303)*.* For reference, in the Data Analyses section we state, *“The dependent variable for listening performance was word recognition accuracy measured as the percentage of 25 scoring keywords correctly repeated per topic with 4 topics per condition (i.e., 4 topics x 25 keywords per topic = 100 keywords scored and averaged per condition).”*

*2.12. Lines 273-275: Provide more information about the piloting.*

**Response:** The piloting was conducted on a small subset of older adults and younger adults using the same experimental task and protocol described in the manuscript. We have clarified this on Page 12 lines 315-318, which now states*, “…piloting of the experimental task, using the protocol described above with a small subset of both younger and older adults, was conducted to ensure that the 0 SNR yielded poorer accuracy scores on the CST than the +4 SNR in both age groups, without reaching floor or ceiling effects.”*

*2.13. Lines 357-363: Isn’t it common practice to use 0.5 as the effect size for calculation? On what basis were the effect sizes chosen?*

**Response:** Thank you for pointing out this important distinction on rationale for the effect size chosen in the power analysis. When using G*Power to estimate sample size for a Repeated Measures ANOVA, within-between interaction, the effect of *f* is used, rather than Cohen’s *d*. Therefore, the effect size of *f* = 0.25 is considered a medium effect. The wording in the manuscript has been adapted for clarity and now reads “*The a-priori power analysis was set to achieve 95% power for detecting a medium effect size of f (0.25) on listening performance and driving performance, at a significance criterion of α = 0.05.”* (See Page 15, lines 407-409).

*2.14. Isn’t it that the signal-to-noise ratio expression is usually in dB?*

**Response:** That is correct that the signal-to-noise ratio is expressed in dB. When we first introduce the two SNR conditions in the Methods section (Page 11, lines 308 and 311), we state that an SNR of +4 dB and an SNR of 0 dB were used. For succinctness, only SNR (rather than dB SNR) was used throughout the rest of the manuscript. We have updated the manuscript to clarify this distinction. Page 11, lines 307 – 311 now reads *“Listening difficulty was manipulated by adjusting the target female talker relative to the background noise to obtain two signal-to-noise ratios (SNR): an SNR of +4 dB (intended to be easier listening and will be reported as +4 SNR going forward for succinctness) and an SNR of 0 dB (intended to be harder listening and will be reported as 0 SNR going forward for succinctness).”*

*2.15. For all the ANOVA analyses, the authors should report the homogeneity of variance results.*

**Response:** We have now modified the data analyses section to explicitly indicate that the homogeneity of variance assumptions were met. Specifically, the manuscript now reads, *“Data met all necessary statistical assumptions for the analyses performed, including homogeneity of variance between groups, as assessed by the Levene’s test for equality of variance (p > 0.5).”* (Page 15, line 401 - 403).

*2.16. Lines 397-399: Any reference or justification for using 3.5 SD?*

**Response:** Winsorizing is a widely accepted and commonly used method for managing outliers. Our justification for using a more conservative 3.5 SD cut-off was to account for the heterogeneity typically observed in older adult populations, thereby limiting the number of outliers that would be removed. We have added this justification to the manuscript (see Page 17, lines 452 - 457), which now states, “*This transformation reduced the influence of outliers while maintaining sampling power that would otherwise be compromised if fully removed. Using a 3.5 standard deviation cut-off is conservative to account for the heterogeneity typically observed in older adult populations and thereby limiting the number of outliers that would be removed. Note, outliers were not due to technological or measurement errors.”*

*2.17. Lines 410-411: Exploratory analyses are not used for testing hypotheses. The authors should adjust the p-value for multiple comparisons.*

**Response:** While this an important consideration, these exploratory analyses investigating any possible associations among experimental measures and hearing, vision, and cognition were not hypothesis driven. Applying a Bonferroni correction to such a large number of comparisons would inevitably result in an extremely small p-value threshold (and a high likelihood of Type 2 error), making it overly stringent, and precluding us from seeing any interesting effects that may be revealed through this rich and comprehensive set of outcome measures. There is also some precedence in the literature to suggest that it is not necessary to apply a p-value correction for multiple comparisons when the data under evaluation are not random numbers but actual observations on nature (See “Rothman K. J. (1990). No adjustments are needed for multiple comparisons. *Epidemiology (Cambridge, Mass.)*, *1*(1), 43–46.”). To ensure complete transparency, we have added to the Introduction, “*A* *secondary goal of the current study was to explore associations among listening and driving performance and measures of hearing, vision, and cognition*.” (Page 5, lines 145 - 146). We have also added the Rothman 1990 citation to the data analyses section upon first mention of the exploratory analyses (Page 18, line 467).

*2.18. Lines 411-413: How did the authors decide that the data is an outlier?*

**Response:** To identify outliers we applied a calculation of the mean plus 3.5*SD and the mean minus 3.5*SD to obtain the positive and negative 3.5 SD cutoff values for each main dependent variable, per condition (e.g., cut-off values for the 0 SNR Dual-Task Rural condition separately from cut-off values for the +4 SNR Dual-Task Rural condition, etc.). If any value fell outside of these cutoff points, the value was changed to the appropriate cutoff point (i.e., winsorized by limiting extreme values). For example, if the -3.5 SD cutoff value for a particular condition was 10% for listening performance, if a participant had a score lower than 10%, that score would be changed to 10%, to be within 3.5 SD for that condition.

*2.19. The result section is very difficult to follow and lacks clarity. It will be easier for the readers to follow if both results and discussion are in the same order.*

**Response:** Thank you for bringing this lack of clarity to our attention; we agree that restructuring these sections would be beneficial.

Therefore, the Results section is now presented as:

Listening Performance

- Listening Performance – Raw Values (accuracy)
- Listening Performance – Proportional Dual-Task Costs (accuracy)
- Listening Performance – Correlations: Raw Values (accuracy)

Driving Performance

- Driving Performance – Raw Values (standard deviation of lane position)
- Driving Performance – Proportional Dual-Task Costs (standard deviation of lane position)
- Driving Performance – Correlations: Raw Values (standard deviation of lane position)

Task prioritization

- Task prioritization: Comparison between listening and driving performance proportional dual-task costs.

The Discussion section is similarly structured as:

- Listening performance; effects of age, driving difficulty, and listening difficulty on dual-task word recognition accuracy (which includes the raw values, dual-task costs, and correlations for listening performance)
- Driving performance; Effects of age, driving task difficulty, and listening difficulty on dual-task driving performance (which includes the raw values, dual-task costs, and correlations for driving performance)
- Task prioritization: Differences in relative dual-task effects on listening vs. driving performance.

We have also added a paragraph to the end of the Data Analyses section to orient readers to the order of results presented in the Results section which states, “*The results section below will first present the data analyses for the listening performance variable of word recognition accuracy, followed by data analyses for the driving performance variable of standard deviation of lane position, and concluding with a comparison of task prioritization between listening and driving performance proportional dual-task costs. The listening and driving performance sections will start with presenting data analyses of the raw values in their original form to allow for direct examination of participant performance, then proportional dual-task costs to allow for examination of the magnitude of any proportional dual-task costs seen and to directly compare proportional dual-task costs for listening to those for driving, and finally correlations between listening/driving performance raw values and hearing, vision, and cognitive measures.*” (Pages 18 and 19, lines 476 - 487).

**3. Responses to Reviewer #1 Comments**

*3.1. It would be helpful if the authors referred to the listening task as the “primary task” and the driving task as the “secondary task,” as these are the commonly used terms in dual-task paradigms. Using this terminology would improve clarity and make the manuscript easier to follow.*

**Response:** Thank you for bringing this to our attention. Although we recognize that traditionally tasks are often referred to as primary and secondary, this is typically under situations when participants are explicitly asked to prioritize one task over the other (or where this is otherwise presumed). We intentionally did not specifically ask participants to prioritize one task over the other or introduce one task as the primary task and one task as the secondary task in our participant instructions. This was intentional as we wanted the paradigm to be more comparable to real-world scenarios for which tasks are not always prioritized in such a way, and prioritization may change rapidly depending on task demands and individual differences. As well, in a real-world listening while driving scenario, we would not typically expect participants to think of the primary task as the listening task and the secondary task as the driving task, due to the safety critical nature of driving. As such, we wanted to see how and whether participants would prioritize one task over the other. We have added a sentence to the Methods section to clarify that participants were not asked to prioritize one task over the other, which states, “*During the dual-task conditions, participants were not instructed to prioritize either task*.” (Page 14, lines 370 - 371).

*3.2. The results section is overly detailed and difficult to follow, even with the use of subheadings. I suggest rewriting it more concisely, focusing on the key outcomes. Additionally, some of the results reported were not clearly linked to the study's stated aims. For instance, the differences in primary task performance across different SNR conditions were not mentioned as objectives. Another example is the association between performance and sensory/cognitive measures, which should be clearly justified as part of the study’s goals. Please revise the section to ensure that all reported results align with the study’s aims.*

**Response:** Thank you for your comments regarding lack of clarity and lack of a clear link between results and study aims; we agree that we could have made our secondary aims more explicit from the outset. Regarding difficulty following the results section, we have adjusted the order of the results to follow the same order as the discussion section. Please see our response in the section 1. Responses to Editors Comments, comment number 2.19.

Relatedly, we have adapted the Introduction, Current Study subsection, to clearly identify that examining task performance across different SNR conditions is part of the primary objectives of the study. This section now states, *“The primary objective of the current study was to examine dual-task costs of listening while driving in younger and older adults with clinically normal hearing using a high-fidelity driving simulator. Further, we also considered the effects of higher vs. lower listening and driving demands on dual-task costs to listening and driving performance.”* (Page 4, lines 133 - 134).

We have also added a sentence to include our secondary goal of exploring the associations among primary outcome measures and sensory and cognitive abilities. Specifically, *“A secondary goal of the current study was to explore associations among listening and driving performance and measures of hearing, vision, and cognition.”* (Page 5, lines 145-146).

*3.3. The authors compared dual-task performance using two different methods: proportional and non-proportional, which may cause confusion for the reader. It would be more effective to choose a single method for assessing changes in dual-task performance and clearly state this in the analysis. This would avoid the need to report results twice for both the primary and secondary tasks which would make the findings easier to follow.*

**Response:** We do understand how using two different methods to examine dual-task costs may initially appear to be redundant and possibly confusing; however, we do think both approaches are very important to include given that proportional and non-proportional dual-task costs offer complementary and distinct insights. Specifically, the non-proportional dual-task costs data are in the original raw form. Examining raw values allows the reader to determine how participants were performing under each of the different conditions presented in their original metrics (i.e., % correct and lane position variability in meters). Additionally, examining raw values allows us to assess whether SNR and/or driving difficulty uniquely influence single-task and dual-task performance, highlighting potential differences in their effects on each task type, which would not be evident if collapsed into proportional dual-task costs. On the other hand, the proportional dual-task costs help isolate the *magnitude* of the dual-task costs and whether this magnitude varies with group, SNR, and driving difficulty. Most importantly, proportional dual-task costs allow us to directly compare listening performance to driving performance by converting them into a comparable metric.

To clarify these aspects to the reader, we have updated the manuscript Data Analyses section to specify what the raw values and proportional dual-task costs are being used for. This is included in the final paragraph of the Data Analyses section that was added to address the comments above (i.e., comment 2.19 and 3.2). Specifically, *“The results section below will first present the data analyses for the listening performance variable of word recognition accuracy, followed by data analyses for the driving performance variable of standard deviation of lane position, and concluding with a comparison of task prioritization between listening and driving performance proportional dual-task costs. The listening and driving performance sections will start with presenting data analyses of the raw values in their original form to allow for direct examination of participant performance, then proportional dual-task costs to allow for examination of the magnitude of any proportional dual-task costs seen and to directly compare proportional dual-task costs for listening to those for driving, and finally correlations between listening/driving performance raw values and hearing, vision, and cognitive measures.”*

We have also changed the dual-task costs subheading in the Results section to “*Proportional Dual-Task Costs…”* to be more explicit about what this section is comparing, and to align with the Data Analyses section more closely. We also added the word “proportional” to the results subheading “*Task prioritization: Comparison between listening and driving performance proportional dual-task costs*” to be more explicit about what this section is comparing.

*3.4. “Raw values – Driving performance (SDLP)”; I do not understand how SDLP is an acronym to this statement.*

**Response:** Thank you for point this out. Similar to the “Raw Values – Listening performance (accuracy)” subheading, SDLP in brackets is meant to reflect the driving performance metric discussed in this section (i.e., standard deviation of lane position). We have spelled out SDLP in each subheading in the Results section for clarity (see lines 595, 632-633, and 665-666). The subheadings now read “*Driving performance – Raw values (standard deviation of lane position)”*, “*Driving performance – Proportional dual-task costs (standard deviation of lane position)”,* and *Driving performance - Correlations: Raw values (standard deviation of lane position)****.***

*3.5. Section starting line 533; I do not understand the significance of this analyses and how is it different from the post-hoc analysis performed in the earlier sections.*

**Response:** Thank you for pointing out the confusion on the significance of the section initially titled “Comparison between listening and driving performance dual-task costs”. To clarify, this section is reporting whether there were any significant differences between proportional dual-task costs to listening performance and proportional dual-task costs to driving performance as a function of group, listening difficulty, and driving difficulty. By directly comparing proportional dual-task costs of listening performance to driving performance, we can better understand task prioritization, which is discussed in the “Task Prioritization: Differences in relative dual-task effects on listening vs. driving performance” in the Discussion Section. We have added the words “Task prioritization:” to the subheading to more clearly articulate the purpose of this section, and to better align the Results section with the related Discussion section. The subheading now reads, *“Task prioritization: Comparison between listening and driving performance proportional dual-task costs”* (Page 27, line 700 - 701).

*3.6. Lines 645-648; Typically, dual-task cost is calculated based on the deterioration of secondary task performance. This statement is somewhat misleading, especially given the significant deterioration observed in secondary task performance in both groups*.

**Response:** Thank you for your comment regarding the statement *“Age-related differences in dual-task costs to word recognition accuracy may be attributable to the observed age-related differences in poorer speech-in-noise thresholds for older adults compared to younger adults (CDTT SRT in Table 1)”*. The first two paragraphs of the Discussion are intended to summarize the listening task performance results, then the driving task performance results. To clarify this, we added a "Summary" subheading at the beginning of the Discussion section (Page 28, line 730). We agree that both listening and driving tasks are crucial to interpreting the results and we address their interplay in the three main Discussion subsections following (e.g., lines 890 - 896)

*3.7. Lines 170-171: what is the degree of hearing loss in the worse hearing ear on this basis for these two participants? Safer to remove these two participants to avoid the risk of the inclusion of participants with unilateral hearing loss.*

**Response:** One participant had a PTA of 35 dB HL in the worse hearing ear, and the other participant had a PTA of 33 dB HL in the worse hearing ear. Since these two participants performed similarly on the other auditory measures collected and the main experimental task, compared to participants with <15 dB interaural difference in PTA, we felt that these participants were not likely skewing the data because of unilateral hearing loss. Therefore, we opted to include these two participants to maintain sampling power that would otherwise be compromised if fully removed. Furthermore, we did not manipulate the spatial location of our listening task stimuli, as all sounds came from all speakers that surrounded the participant in all directions. Therefore, any potential effects of asymmetry on listening task performance would be minimal.

*3.8- I suggest the authors arrange the discussion following the same order used in the results section. E.g. association between performance in the dual task and different measure of speech in noise cognition etc. is reported at the end of the results section but discussed early in the discussion.*

**Response:** Thank you for your suggestion on improving the overall clarity and flow of the manuscript Results and Discussion sections. We have adjusted the order of the results to follow the same order as the discussion section. Please see our response in Section 1, Editors Comment 2.19.

*3.9. When discussing the association between performance on the primary and secondary tasks with measures such as speech-in-noise, cognition, and vision (e.g., in the paragraph starting on line 718), please clarify whether the results pertain to the older group, the younger group, or both.*

**Response:** Thank you for pointing out this important clarification point. We have updated the Data Analyses section to clarify this distinction and it now states, “*A series of Pearson’s correlations were performed across all participants to examine whether there was a relationship between listening and/or driving performance experimental measures with vision, hearing, and/or cognitive baseline measures.”* (Page 18, line s463 - 465).

*3.10. Lines 111-112; worth adding a comment of how this represents real life situation where sometimes roads can be noisy making listening difficult.*

**Response:** Thank you for bringing up this great point. As you note, in the context of driving, a noisy environment can be introduced through various sources, such as road noise, which can make listening difficult. We acknowledge this on Page 2, line 79 - 82, where we state, “*The complex task of driving reflects real-world acoustical challenges (e.g., background noises, reverberation), other challenging environmental conditions (e.g., poor weather) and perceptual challenges to other sensory modalities (e.g., reduced visual inputs at night), and requires coordinating multiple sensory, cognitive, and motor tasks simultaneously*.” We feel this is an appropriate place to make this important point.

*3.11. Line 152; so they are not all natives? how frequently was English used in there life then? although some can learn English at young age and speak it fluently, but that does not make it as strong as there mother language.*

**Response:** While we do not know whether English is their native language or how frequently they speak English, we do know that they all learned English as young children and that for the vast majority (all but 2), English was their primary language of choice. (See also responses to Editor comment 2.1. We have added these additional details to the manuscript (Page 5, lines 156 - 158) which now reads “All participants provided written informed consent, spoke fluent English, learned English by the age of 5, and *reported English as their primary language of choice (with the exception of one younger and one older adult participant) .”*

*3.12. Lines 405-406; please provide a reference.*

**Response:** Thank you for your comment regarding the statement “*A value of zero would indicate neither a dual-task cost nor a dual-task benefit, therefore, any significant differences between proportional dual-task values and zero would indicate significant dual-task costs/benefits.”* To our knowledge, there is no appropriate reference to cite when discussing what a value of zero means regarding dual-task costs/benefits, as this is common practice in the dual-tasking literature (for example, see Somberg and Salthouse 1982 for a rationale on the use of proportional dual-task costs in aging studies: Somberg, B. L., & Salthouse, T. A. (1982). Divided attention abilities in young and old adults. Journal of Experimental Psychology: Human Perception and Performance, 8, 651–663). A value of zero would mean that performance in the single task is not different from performance in the dual task, thus, no dual-task costs/benefits occurred. As such, if the difference between single and dual-task performance significantly differs from zero, there would be a change in performance from single to dual task, and the direction of that change would indicate a cost (positive value) or benefit (negative value).

*3.13. Lines 742 to 747, I do not understand the argument that the authors are trying to make here. Are they referring to their own work? Please clarify.*

**Response:** Thank you for your comment regarding the statement, “*Most previous dual-task driving studies have implemented an auditory secondary task to introduce overall cognitive demands rather than strategically selecting an auditory task that may be sensitive to known age-related changes in hearing, such as speech-in-noise tasks. During conversational tasks in quiet conditions, older adults often perform similarily, if not better than younger adults, due to the use of contextual speech cues and vast linguistic knowledge.”* In this section we are speculating about why the present study findings showing age-related differences in dual-task costs are different from several previous dual-task driving studies (that are not our own) that demonstrated no age-related differences. We have revised this section to more clearly outline the various factors contributing to the differences in findings between the current study and previous driving literature. This section now states, “*One potential reason for these discrepancies could be due to the type of auditory tasks used in previous studies. Specifically, most previous dual-task driving studies have implemented an auditory secondary task to introduce overall cognitive demands rather than strategically selecting an auditory task that may be sensitive to known age-related changes in hearing, such as speech-in-noise tasks. Another contributing factor may be differences in the availability of contextual speech cues and vast linguistic knowledge across studies. Older adults often perform similarly if not better than younger adults when such cues are present (56), with their linguistic expertise potentially reducing cognitive demands compared to younger adults.”* (Page 32, lines 940 - 947).

*3.14. Lines 759-762, again, how is this related to the previous paragraph? Are the authors referring to their own study? Please clarify?*

**Response:** The previous paragraph simply describes the associations between the driving measures observed in the current study with basic baseline measures (i.e., hearing, vision, cognition) and how these associations may be used to interpret the primary dual-task outcomes observed in the current study and discrepancies in findings from previous literature as it relates to the listening tasks used. The next paragraph builds on this discussion by focusing on how these associations might also clarify inconsistencies in the broader literature, particularly regarding the types of driving tasks used. While we note that previous studies using highly complex driving tasks do not find age-related differences in dual-task costs to driving performance, the lines in question (lines 759–762) emphasizes that fully understanding these findings requires considering both driving and auditory task performance. To clarify that we are referring to previous work, we have added the words “in previous studies” to Page 33 lines 972 – 974, which now states, “*However, it is difficult to make clear interpretations on the effects of dual-tasking on driving performance in younger and older adults in previous studies because the complexities of both the driving task and the listening task need to be considered*.”

Additionally, we have revised the opening of the final paragraph in this section (i.e., Effects of age, driving difficulty, and listening difficulty on dual-task driving performance*)* to establish a clearer connection to the preceding paragraph, which now states:, “*Associations between driving performance and sensory/cognitive measures observed in the present study may also help explain findings from previous driving studies, particularly in relation to the complexity of driving tasks employed. While the variety* *of driving tasks that are often not clearly described* *in previous dual-task driving studies make it difficult to categorize driving task difficulty levels, the studies that do provide clear descriptions consistently report no effects of age on dual-task costs to driving performance, regardless of whether the dual-task driving studies used simplistic driving scenarios that were relatively easier (e.g., driving straight down a two-lane road with minimal amounts of buildings and traffic; (16,17,19,52,53)) or relatively complex (e.g., driving through intersections with many buildings and high levels of traffic; (18,51)).”* (Page 33, lines 963 – 972).

*3.15. Sentence in line 792 contradicts the rest of the paragraph.*

**Response:** Thank you for pointing this out. The final sentence in this paragraph stating, “*the dual-task costs to driving overall may indicate a general increased risk of compromised safety*”, is meant to touch on the fact that although we did not directly measure driving safety, poorer driving performance when dual-tasking compared to single tasking could potentially result in a greater risk of compromising driving safety. The key part of this statement is “increased risk”. We have italicized the words *increased risk* in the manuscript to better reflect our intentions (Page 34, line 1013).
