# Supplementary material for: Dual-task costs of listening while driving in older and younger adults
Source: PLoS One. 2025 May 29;20(5):e0324657. doi: 10.1371/journal.pone.0324657 (PMC12121817; doi:10.1371/journal.pone.0324657)
Supplement: Attachment — Submitted filename: Response to Reviewers_Round 2.docx [file pone.0324657.s010.docx]

We would like to sincerely thank the editor and reviewers for taking the time to provide additional thoughtful comments towards improving our manuscript. This document lists all comments specific to each reviewer, interleaved with the authors’ responses and, when applicable, references to the changes made to the manuscript. We are confident that these changes have improved the manuscript and we hope that you will again consider it for publication in PLOS ONE.

**1. Responses to Editor Comments:**

*1.1. Lines 144-145, “A secondary goal of the current study was to explore associations among listening and driving performance and measures of hearing, vision, and cognition”: The authors have introduced these two lines without a proper context in the introduction. Reviewer-1 has raised similar concerns.*

**Response:** Thank you for pointing this out. We have added some additional context to the introduction of the manuscript to justify the inclusion of our secondary objectives, which can be found on Pages 3 and 4 lines 113 to 118 and states: “*Previous literature has shown associations between hearing and cognition in healthy older adults (Lin, 2011, 2013). However, to our knowledge few experimental studies have examined how hearing measures (e.g., pure-tone threshold average [PTA], words-in-noise test scores) and cognitive measures (e.g., working memory, executive control) are independently associated with performance on more complex listening-while-driving dual-tasks.*”

We also removed vision measures from the secondary objective, as the main motivation for this secondary goal comes from previous literature examining the link between hearing and cognition specifically. While we acknowledge that driving is a highly visual task, and that basic vision tests might be associated with dual-tasking performance, our only measure of vision was visual acuity. Since participants typically correct for reduced visual acuity with prescription glasses, the range of visual abilities that could be assessed was limited. Notably, visual acuity showed few to no significant associations with our primary outcome measures. Therefore, vision has been excluded from the correlation analyses and related figures.

*1.2. Hearing testing: Based on what the authors have mentioned, it is not a screening test, the authors have conducted an air-conduction hearing test. Figure 1 clearly illustrates that hearing thresholds in older adults are elevated compared to those of younger adults. However, it is important to note that bone conduction thresholds are not included in the audiogram. This raises the question: Could the older participants have slight sensorineural hearing loss? According to the WHO (1991) guidelines on the grades of hearing impairment, individuals with thresholds between 15-20 dB may experience hearing difficulties. Figure 1 indicates that some individuals in the older group had elevated hearing thresholds at 3 and 4 kHz, which could have contributed to their reduced performance on these tasks. Are we underestimating the impact of these elevated thresholds on complex dual-task performance? Hearing thresholds at high frequencies play a crucial role in recognizing speech in challenging listening environments. Consider adding these thresholds as a covariate in the analyses to assess if they have any impact.*

**Response:** Thank you for your insightful comment. We fully agree that elevated high-frequency pure-tone thresholds may play a role in speech understanding in challenging listening environments. Our goal, however, was not to minimize the influence of these age-related changes, but rather to note their relevance and importance as a motivation for this study (e.g., Page 1 lines 48-53, Page 3 lines 108-113, and Page 4 lines 128-132). It is important to note that this study specifically excluded older adults with pure-tone thresholds exceeding clinically significant levels (average of ≥25 dB HL in the better ear across 500, 1000, 2000, 3000, and 4000 Hz, the main speech frequencies). We intentionally included older adults with the commonly observed elevated thresholds (relative to younger adults). This was intentional to be able to examine how age-related changes in hearing abilities affect dual-task performance. Indeed, one of the aims of conducting correlations between primary outcome measures (e.g., driving performance) and baseline sensory measures, such as pure-tone thresholds, was to investigate the potential associations.

Upon further review, we identified a section in the Discussion that did not adequately address the potential influence of pure-tone thresholds on listening performance. We have now clarified this in the Discussion Summary (Page 27 lines 857–860), which states: “*Age-related differences in dual-task costs to word recognition accuracy may be attributable to the higher speech-in-noise thresholds and elevated high-frequency pure-tone thresholds for older adults compared to younger adults (CDTT SRT and PTA Better Ear respectively, Table 1).”*

*1.3. Additionally, the authors referenced the WHO (1991) guidelines for calculating hearing thresholds at 0.5, 1, 2, 3, and 4 kHz frequencies. However, the table in the guidelines specifically recommends calculating hearing thresholds only at 0.5, 1, and 2 kHz. The authors have to clarify this.*

**Response:** Thank you for bringing this to our attention. We have clarified that we used a modified version of the WHO guidelines, on Page 6 line 180, by stating: “*All participants had clinically normal hearing, defined as an average of 25 dB HL or less in the better ear across the five frequencies tested (criteria adapted from 30,31) apart from one younger adult whose audiometric data did not save due to technical issues but this participant self-reported normal hearing.”*

We decided to include 3 and 4 kHz to provide a more comprehensive representation of the speech frequency range compared to the WHO criteria (Hughes, G. W., & Halle, M. (1956). Spectral properties of fricative consonants. *Journal of the Acoustical Society of America*. https://doi.org/10.1121/1.1908271), which is focused more on clinical applications of this criteria. We would also like to note that if calculations were done excluding the 3 kHz frequency, all participants would still be considered to have clinically normal hearing (i.e., PTA less than or equal to 25 dB HL in the better ear).

*1.4. The authors do not clearly state the calibration procedure which is an important part of this manuscript if someone wants to replicate it.* *Lines 284-285: What sound pressure levels were measured? To determine the sound pressure level, what type of sound was used? Was it the target sentence or the background noise? How many times did these calibrations were conducted?*

**Response:** The sound pressure levels were measured for the CST babble and the DriverLab environmental and vehicle noises (e.g., mixture of engine noises, tires rumbling on the pavement, A/C fans). The duration of a sample was 8 minutes (the time it took for a driving scenario). These measurements were conducted on 3 samples to obtain the average sound level (dBA) reported in the manuscript. We have further clarified this procedure in the manuscript on Pages 11 and 12, lines 300-312, which now states: “*The CST was presented through all DriverLab speakers. The sound levels were measured using the REED R8050 SLM and were, on average, consistent across drives. Specifically, background noise, including the CST babble and DriverLab environmental and vehicle noises (e.g., engine noises, tires rumbling on the pavement, A/C fans) were measured three times, with an average of 64 dB A across all conditions. Listening difficulty was manipulated by adjusting the target female talker relative to the background noise to obtain two signal-to-noise ratios (SNR): an SNR of +4 dB (intended to be easier listening) and an SNR of 0 dB (intended to be harder listening).”*

Information about the calibration procedure of the SLM was included in the previous round of revisions and can be found in the Apparatus section of the Methods (Page 11, lines 284-286). To clarify, the SLM was calibrated before each use.

*1.5. I think it is appropriate to report SNR in dB throughout the manuscript, consistently.*

**Response:** We have added dB to all mentions of SNR throughout the manuscript.

*1.6. In the previous revision, I asked about the piloting process. How was it conducted, and how many participants were involved? Additionally, how many participants participated in both the pilot and the main study? If some participants were involved in both, they would have been familiar with the study. Do the authors believe that this familiarity with the study and the tasks could have influenced the results?*

**Response:** We now recognize that the wording describing the piloting procedure may have been misleading. The pilot participants were not a subset of the main study sample, but rather entirely different participants who never participated in the main study. Before launching the main study, piloting of the procedure and stimuli was conducted on 6 younger adults and 4 older adults. Thus, there were no familiarity or carry over effects between piloting and the main study. We have further clarified this in the manuscript, Page 12 lines 314-318, which now states: “*Therefore, piloting of the experimental task, using the protocol described above with a small sample of younger (N=6) and older (N=4) adults, was conducted to ensure that the 0 dB SNR yielded poorer accuracy scores on the CST than the +4 dB SNR in both age groups, without reaching floor or ceiling effects. Participants involved in the piloting phase did not participate in the main study.”*

*1.7. The authors have responded to my question, stating that "it is not necessary to apply a p-value correction for multiple comparisons when the data under evaluation are not random numbers but actual observations of nature" (Rothman K. J., 1990, Epidemiology). However, I find their response regarding p-value adjustments for multiple comparisons unconvincing. If the authors do not support adjusting the p-value, they should refrain from using it altogether in this manuscript. The authors have applied a Bonferroni correction in the manuscript (lines 378-379), but this is not the only available method. The False Discovery Rate (FDR) is another appropriate approach the authors could consider.*

**Response:** Thank you for bringing this to our attention and for offering an alternative approach. We agree that it is often beneficial to apply consistent parameters and statistical criteria throughout the results section. We also recognize that supporting the use of multiple comparison corrections for some analyses while not implementing them for others may appear inconsistent. However, we would like to clarify that different analyses in this manuscript serve distinct purposes and, therefore, require different strategies. Specifically, the ANOVAs conducted on listening and driving performance are the primary outcome measures, driven by our hypotheses and the main focus of the paper. As such, we opted for a more conservative approach by applying the **Bonferroni correction** for multiple comparisons, which helps reduce the likelihood of Type I errors (i.e., detecting an effect that does not actually exist). On the other hand, the correlations between listening and driving performance and baseline measures of sensory and cognitive abilities are exploratory in nature and secondary to the primary goals of the study. Since these correlations were not hypothesis-driven, and there were many comparisons, we chose to transparently implement a more liberal approach by not applying a correction for multiple comparisons. This strategy reduces the likelihood of Type II errors (i.e., failing to detect a true effect). If we had applied the Bonferroni correction to these exploratory correlations, the required p-value to detect an effect would have been extremely small (approximately 0.00083) due to the large number of comparisons being conducted.

While the **False Discovery Rate (FDR)** approach allows for more discoveries than stricter corrections like Bonferroni, it still involves a threshold that could prevent the detection of significant effects, especially when those effects are subtle. We hope that these exploratory analyses will help inform future research that can apply more stringent criteria to directly test these effects. Moreover, since we have a wealth of baseline data that can be analyzed in the context of the dual-task paradigms used in the current study, these exploratory analyses were intended to maximize the use of this rich dataset to gain deeper insights into the relationship between sensory and cognitive measures and our primary outcome measures. Additionally, in the discussion section, we reference Table 1, which uses **t-tests** to examine group differences in baseline measures (e.g., Independent Samples t-tests which found significant differences between younger and older adults for PTA and CDTT SRT). This helps provide additional evidence and context to support the exploratory correlation results we report using a more conservative approach.

We also explicitly explain to readers that these analyses are exploratory, and no corrections for multiple comparisons were applied with full transparency, indicating that these results should be interpreted with caution. We have added statements in the Discussion on Page 28, lines 894-896, and Page 31, lines 959-961, clarifying this point: “*It is important to acknowledge that these associations are exploratory in nature, and no corrections for multiple comparisons were applied. Consequently, these findings should be interpreted with caution*.”

*1.8. The results section remains difficult to follow. The authors should focus on presenting the key findings that they want the readers to take away, ensuring clarity and emphasis on the most important messages.*

**Response:** We acknowledge that the results section was previously lengthy and potentially challenging to navigate, as much of the findings presented in the raw values were consistent with those reported in the proportional dual-task costs. To improve the overall flow and clarity of the manuscript, we have moved the detailed analyses and figures for proportional dual-task costs to the Supplementary Materials. As a result, the results section now includes five figures instead of seven, making it more concise and easier for readers to focus on the key findings.

We still defend including the proportional dual-task costs in the Supplemental Materials rather than removing them all together given that this is a very common approach typically expected for dual-task studies and most importantly because they do offer additional insights. Specifically, proportional dual-task costs provide unique information regarding whether the differences observed between single- and dual-task conditions are significantly greater for one group compared to the other. While the raw values offer valuable information, they do not directly address the magnitude of these differences between groups—an essential finding we aim to highlight. To ensure these unique findings are accessible to readers, we have included a brief paragraph at the end of the Listening Performance – Accuracy and Driving Performance – Standard deviation of lane position (SDLP) sections referencing the Supplementary Materials, summarizing the key unique insights from the proportional dual-task costs without reporting the full analyses. Lastly, determining *task prioritization/relative task costs* to listening and driving performance is only possible by referring to the proportional dual-task costs.

The Results Section is now presented as:

**Listening Performance – Accuracy**

- Correlations (listening accuracy)

**Driving Performance – Standard deviation of lane position (SDLP)**

- Correlations (standard deviation of lane position)

**Relative Costs to Listening and Driving Performance**

- Comparison between listening and driving performance proportional dual-task costs.

**2. Responses to Reviewer #1 Comments**

*2.1. I still find this manuscript lengthy and challenging to follow. The extensive analyses, lengthy results section, and inclusion of 11 figures make it a difficult read. I strongly suggest that the authors consider streamlining the analyses by focusing on a single method that effectively conveys the main message of the manuscript. This would improve clarity and readability. Additionally, I am not entirely convinced that proportional analyses serve as an appropriate measure of prioritization in the context of the task at hand.*

**Response:** Thank you for this note. We have now adapted a significantly streamlined version of the results section. Please see our response to the Editor’s Comment #1.8 above. We have also changed “Task Prioritization” to “Relative Costs” to address concerns about the previous framing, which we agree required finetuning. Please see our response to comment #2.9 below.

*2.2. Based on this, I am not entirely confident that the term "listening effort" accurately describes what is being assessed in this context. It is difficult to imagine a scenario where the quality of driving is compromised in order to prioritize listening. Since driving quality pertains to a safety-critical issue, it is highly unlikely that listening would ever be treated as the primary task. As I previously mentioned, in the literature on listening effort, listening is typically regarded as the primary task that consumes the majority of cognitive resources, with any other task being considered secondary. This framework applies even when participants are explicitly instructed to prioritize either the primary or secondary task. Therefore, I believe the author needs to provide a stronger justification for why the measured construct should be considered "listening effort" rather than, for example, "driving effort."*

**Response:** We agree with the reviewer and that is why we do not use the term “listening effort” when describing our specific objectives, methods, and discussion of the current study findings. The primary aim of this study was to understand how older and younger adults allocate cognitive resources during a listening-while-driving task, rather than specifically focusing on the construct of listening effort per se. Throughout the paper, particularly in the Discussion section, we try to be careful when interpreting the findings, referring to “increased listening difficulty” (which we manipulate through SNR) and “driving difficulty” (manipulated through driving task and environment complexity) and the relative effects on cognitive resource allocation. We examine how these demands influence both listening and driving task performances together, rather than attributing the effects solely to increased listening effort per se. For instance, we use phrasing such as “increased listening difficulty may have resulted in more resources being allocated to listening, thereby reducing the cognitive capacity available for driving,” which is inferred from patterns in both tasks.

Although it might seem intuitive that driving is always the primary task, this assumption is called into question by the many studies and anecdotal evidence demonstrating that driving while distracted can lead to significant, and at times, catastrophic consequences to driving outcomes. Driving often feels automatic and highly practiced, leading individuals to sometimes become complacent, focusing less on driving and redistributing cognitive resources towards listening in ways that are disadvantageous. There is a vast literature surrounding distracted driving, particularly in the context of auditory distractors, which is briefly described in the Introduction.

*2.3. “We have also added a sentence to include our secondary goal of exploring the associations among primary outcome measures and sensory and cognitive abilities. Specifically, “A secondary goal of the current study was to explore associations among listening and driving performance and measures of hearing, vision, and cognition.” (Page 5, lines 145-146).”
Inserting this to the end of the introduction in this way comes out of nowhere as it his hard for reader to understand the motive for this investigation given that related literature was not addressed in the introduction.*

**Response:** Thank you for this note; we agree that more contextualization was required. We have now added more information to the Introduction section to articulate the motivations more clearly for investigating these relationships. Please see our response to the Editor’s Comment #1.1 above.

*2.4.  Lines 144-145; it is good that the authors have added this as a goal of the study, however, the rationale for exploring these association and the implications of doing so should have been introduced to the reader somewhere in the introduction of the manuscript.*

**Response:** Please see our response to the Editor’s Comment #1.1 above.

*2.5.  Line 155; please remove the word “and” from the end of the sentence “and reported”.*

**Response:** Thank you for pointing out this grammatical error. We have removed the word “and” from the indicated section on Page 5 line 160, which now states: “*All participants provided written informed consent, spoke fluent English, learned English by the age of 5, reported English as their primary language of choice (with the exception of one younger adult and one older adult participant), had a valid driver’s license with at least two years of driving experience…”*

*2.6. Line 177; add what you mentioned in the rebuttal letter here about these two participants.*

**Response:** We have added further information noted in the rebuttal letter to the manuscript on Pages 6 and 7 lines 190-197, which now states: “*The two participants who had interaural differences between PTA scores >15 dB performed similarly on these other auditory measures and the main experimental task, compared to participants with <15 dB interaural difference in PTA scores. Therefore, these participants were not likely skewing the data. We decided to include these two participants to maintain statistical power.*

*2.7. Line 209; mention what they were used for, i.e. some further analysis to address the research questions later? Same applies to wherever the same statement was mentioned in the methods section.*

**Response:** Thank you for pointing this out. We have further clarified why these analyses were conducted by stating “...*examine exploratory associations with primary outcome measures*” when describing analyses that involved the other baseline measures that were not used to determine eligibility, but were rather used to characterize the sample (i.e., CDTT SRT, Digit Span, Trail Making Test, Stroop, UFOV). These changes have been made on Page 6 lines 189-190, Page 8 lines 223-224, and Page 8 lines 230-231.

*2.8- Line 347: Not clear how? What were the instructions exactly?*

**Response:** Participants were not instructed to prioritize either task. Instead, participants were asked to complete two tasks at the same time. Specifically, they were instructed to drive safely and follow the lead car while also repeating back the sentences as best as they could. The manuscript has been updated to reflect these task instructions more clearly on Page 14 lines 375-378 and now states: “*During the dual-task conditions, participants were asked to complete the two tasks at the same time. Specifically, participants were asked to drive safely and follow the lead car while also repeating back the sentences as best as they could. Participants were not instructed to prioritize either task.”*

*2.9. “The current study found that both older and younger adults had greater proportional dual-task costs to driving compared to listening. Specifically, for older adults, this pattern of prioritization was only observed under the most difficult listening and driving conditions.”
Need to explain how proportional analysis addresses the concept of task prioritization more explicitly.*

**Response:** Proportional analyses allow for the direct comparison between the relative dual-task costs to listening performance and relative dual-task costs to driving performance. This normalization procedure allows these metrics to be directly comparable on the same scale. Upon further reflection and based on your helpful feedback, we recognize that the term “task prioritization” may not accurately capture the intended interpretation of this outcome. Instead, this outcome reflects *relative costs* to driving and listening without making any assumptions about prioritization. Our findings demonstrate that both older and younger adults had greater proportional dual-task costs to driving compared to listening. This does not imply, however, that listening was prioritized over driving, simply that the costs of performing two tasks at the same time had a greater influence on driving than listening. Therefore, to better capture the nature of this comparison, we have revised the terminology, replacing “task prioritization” with “relative task costs to driving versus listening” throughout the manuscript. This includes changes to subsection headings in the Results and Discussion sections, as well as within the main body of the Discussion text.

*2.10. For younger adults, greater dual-task costs to driving compared to listening were only observed under conditions when listening was easier (+4 dB SNR) and driving was harder (City), for which there were dual-task costs to driving performance but dual-task benefits to listening performance”. Why is this explained as prioritization? Could it not simply be that one task is easier than the other? In dual-task paradigms, participants often tend to prioritize the easier task. In this particular case, this might be especially true given that it involves a simulated driving scenario, where participants are aware that there is no real risk of harm.*

**Response:** Thank you for this comment. This section focuses on the relative differences in dual-task costs between listening and driving performance for younger and older adults. We believe the revisions outlined above (comment #2.9) regarding the use of "task prioritization" will help clarify this point. As you noted, the relative difficulty of the two tasks is also important to consider. The final paragraph of this section (Pages 33 and 34, lines 1026-1040) discusses how dual-task costs relate to task difficulty and contextualize our results within the inverted U-function of task demands and performance.

The concern about the validity of driving simulation in this context (e.g., no fear of real risk) is also a potential limitation of simulator research in general; however, there is empirical evidence to support the validity of driving simulators. For example, research on the validity of driving simulators shows evidence of a strong sense of presence even in the absence of real physical harm, through heart rate and electrodermal activity (Walker et al., 2019 – https://doi.org/10.1504/ijhfms.2019.10027090), and similar braking reaction times to a potential crash between simulator and on-road performance (McGehee et al., 2000 - https://doi.org/10.1177/154193120004402026). DriverLab is also the most advanced high fidelity driving simulator in Canada, and among the top five most advanced in the world, enhancing the realism and immersive quality of the experience. Anecdotally, many of our participants in this study described the driving simulator as seeming highly realistic. Participants instinctively checked their blind spots and displayed outward signs of heightened arousal when faced with challenging scenarios, such as when approaching a yellow light, providing further evidence of a sense of perceived risk.

*2.11. 893-894: what do you mean by contextual speech cues?*

**Response:** We do not see any mention of contextual speech cues on lines 893–894. Perhaps you are referring to line 785, which states: 'Another contributing factor may be differences in the availability of contextual speech cues and vast linguistic knowledge across studies.'? Here, we are referring to the fact that most previous dual-task driving studies have used auditory secondary tasks lacking the contextual cues and information present in natural speech situations, which the current study’s use of the Connected Speech Test incorporates. This may help to understand differences in results between our study and previous literature. Contextual speech cues are elements within speech that help listeners interpret meaning by providing additional context. For example, semantic context allows words and phrases to provide meaning based on their relationship to one another, helping listeners predict upcoming words. Another relevant example is *topic continuity*, where previously mentioned information about a topic guides the listener in interpreting subsequent words or sentences.

To clarify this point, we have modified the sentence to specify what we mean by contextual speech cues. Page 31 lines 970 to 972 now reads: 'Another contributing factor may be differences between studies in the availability of supportive linguistic context, including lexical, syntactic, and semantic cues within and between sentences.'
