# Supplementary material for: Dual-task costs of listening while driving in older and younger adults
Source: PLoS One. 2025 May 29;20(5):e0324657. doi: 10.1371/journal.pone.0324657 (PMC12121817; doi:10.1371/journal.pone.0324657)
Supplement: Attachment — Submitted filename: Response to Reviewers_Round 3_V3_Apr 17 2025.docx [file pone.0324657.s011.docx]

We would like to sincerely thank the reviewer for taking the time to provide additional thoughtful comments towards improving our manuscript. This document lists all comments, interleaved with our responses and, when applicable, references to the changes made to the manuscript. We are confident that these changes have improved the manuscript and we hope that you will again consider it for publication in PLOS ONE.

**1. Responses to Reviewer #1 Comments**

*1.1. Line 675: There is a typographical error in the word "adults."*

**Response:** Thank you - we have corrected the error in the revised manuscript.

*1.2. Lines 676–679: Please provide a clearer and more valid explanation for this observation.*

**Response:** Thank you for pointing out the ambiguity in our description of the study findings. We have revised the manuscript to present the word recognition accuracy results more clearly. Page 28, lines 695-700, now states: *“The current study found that younger adults’ word recognition accuracy was similar across conditions and was not affected by driving difficulty or dual-tasking. However, for older adults, word recognition accuracy was poorer in the more difficult driving condition (i.e., City) compared to the listening only condition, regardless of listening difficulty (i.e., +4 dB SNR and 0 dB SNR). Further, older adults’ word recognition accuracy was also poorer during the easier driving condition (i.e., Rural) compared to the listening only condition when listening was less difficult.”*

*1.3. Lines 682–683: The sentence "There were also some (albeit inconsistent) indicators of associations between word recognition accuracy and poorer performance" is unclear. It is not evident whether an association was found. Please specify the exact findings more clearly.*

**Response:** This sentence was meant to convey that associations among word recognition accuracy and other sensory and cognitive measures were found only for some dual-task conditions, but importantly, *not* in any single-task conditions. We had added “albeit inconsistent” because these associations were not found for every dual-task condition, but we understand that this may have caused confusion. To convey this more clearly, we have revised the sentence on Page 28, lines 702–706, which now states: “There were also some associations between word recognition accuracy and poorer performance on the other sensory and cognitive measures (e.g., PTA, MoCA, Trail Making, Digit Span, UFOV) under some dual-task conditions, but no associations were observed for any single-task conditions (see Figure 6).”

*1.4.  Lines 730–733: I recommend the authors elaborate further on this point. In the listening effort literature, pure-tone average (PTA) is often found to be a poor indicator of listening effort and disability when compared to measures such as speech-in-noise performance and self-report tools (see Alhanbali et al., 2018).*

**Response:** Thank you for this comment. We agree that pure-tone average (PTA) may not be a reliable indicator of listening difficulties experienced by older adults under noisy listening conditions. This is why we differentiated the associations of CDTT SRT with driving performance from the associations between PTA and driving performance. In response, we have expanded this discussion in the manuscript by more explicitly linking previous literature to the present findings. Page 30, lines 762-771, now states*: “For example, better speech-in-noise thresholds (i.e., CDTT SRT) were associated with better driving performance across almost all driving conditions. Notably, audiometric thresholds (i.e., PTAs) were not associated with driving performance under any condition. PTAs may not be a reliable indicator of speech understanding abilities in noisy environments, as older adults with clinically normal audiometric pure-tone thresholds often still perform more poorly on speech-in-noise tests than younger adults with normal hearing (Dubno 1984; Fullgrabe 2015). The current findings appear to extend prior findings to more complex driving tasks, whereby better speech-in-noise thresholds rather than audibility (lover PTA) are associated with better management of the relative demands of performing a speech-in-noise word recognition task while driving.”*

*1.5.  Lines 767–789: I suggest either removing this section or substantially shortening it. It is currently difficult to follow, and its relevance to the rest of the discussion is unclear.*

**Response:** Thank you for this suggestion. The final paragraph originally addressed how discrepancies in previous literature may have potentially resulted from differences in driving task complexity. The preceding paragraph focused on similar discrepancies, but in relation to listening task differences. It also used current findings on associations between sensory and cognitive measures and driving performance to help interpret prior research. Upon further reflection, we have combined these two paragraphs into a single, more concise discussion that integrates both points and streamlines the content. We hope these changes make this section easier to follow, and that the relevance to the rest of the discussion is clearer. Please see Pages 31-32, lines 790-825.

*1.6. Lines 832–838: The argument presented here is not entirely convincing. Although older adults may have more driving experience, age-related declines in cognitive, motor, and other functions are likely to offset the benefits of experience.*

**Response:** While the main objective of the study was to evaluate age-related differences in dual-task driving performance, driving history and experience is essentially confounded with age and age-related sensory/motor/cognitive changes. Specifically, older adults in our study had, on average, over 49 years of driving experience, compared to only 9 years of driving experience in younger adults. It is very likely that both age-related declines in sensory/cognitive/motor functioning *and* experience both contributed to the driving outcomes observed in this study, with the relative contributions of each remaining unknown, but acting in opposite directions. Also notable is that older adults in our study were also very active, healthy, participants who were screened for any significant sensory or cognitive declines or impairments. As such, the negative effects of any milder age-related declines may have been less impactful than what might be expected from the more general older adult population who have various impairments and comorbidities (as we already address as a limitation). As such, it might be expected that such an extensive history of driving experience combined with generally mild declines, may have allowed experience to play a compensatory role, particularly in the easier driving conditions (i.e. Rural), for which we indeed saw no age-related differences in the results. We have thus adjusted this section to address these considerations more directly, which now reads on Page 34, lines 906-911: *“The older adults in this study had significantly more years of driving experience (M= 49.05 years) than younger adults (M=9.88 years). Age-related differences in driving experience between age groups in the current study may have reduced the age-related effects on some of our outcome measures, particularly under the easier driving conditions (Rural) because older adults could compensate for potential age-related declines in sensory/cognitive/motor abilities by relying on well-practiced driving skills and expertise.”*

*1.7. Conclusion section: The recommendation for hearing aid use does not seem appropriate in the context of this study, as the participants all had normal hearing.*

**Response:** Thank you for this note. We have removed the mention of hearing aids from the sentence. Pages 35-36, lines 946-951, now states: *“Future research could examine ways to potentially mitigate these dual-task effects by, for example, working memory training to help reduce the cognitive load associated with speech understanding during driving, optimization of the vehicle acoustics or use of technology to improve the SNR, or minimization of auditory distractions, especially when driving situations become more complex (e.g., in the City).”*
